# Least squares collocation method in Moho depth determination in Iran using gravity gradient data

**DOI:** 10.1016/j.heliyon.2024.e24596

**Published:** 2024-01-23

**Authors:** Hadi Heydarizadeh Shali, Carlo Iapige De Gaetani, Riccardo Barzaghi, Sabah Ramouz, Abdolreza Safari, Barbara Betti, Zohreh Abbasi Hafshejani

**Affiliations:** a Center for Earthquake Research and Information, University of Memphis, Memphis, TN, USA; b Dept. of Civil and Environmental Engineering, Politecnico di Milano, Milan, Italy; c University of Tehran, School of Surveying and Geospatial Engineering, Tehran, Iran

**Keywords:** Collocation, Moho, Gravity inversion, Isostasy

## Abstract

In this study, an approach using gravity observations utilizing the Least Squares Collocation (LSC) method is developed with the aim of mapping the depth spatial variability of the Mohorovičić discontinuity. This approach is based on a spherical two-layer isostatic model where the exterior gravity field only varies because of the shifting topographic masses and the related isostatic adjustment since it is believed that the Earth's core has a uniform density distribution. Assuming mass conservation between the Moho column of height δR with respect to $R_{m}$ representing the mean Moho and following a Helmert condensation approach, the relationship between the surface layer density to the potential δT can be obtained and δR can be estimated via LSC from observed values of any functional derived from δT. With such approach, the depth of Moho in the Iranian Plateau is estimated from $T_{rr}$ data generated by GOCO06S model reduced by topography, bathymetry and sediments effects by considering GEBCO2021 and CRUST1.0 models. The needed a-priori assumptions on $R_{m}$ and the density contrast Δρ are tuned so to obtain the best fit with seismic Moho depths reported by literature. 73 stations were matched with 3 km of standard deviation, which is coherent with the expected accuracy of the benchmark values. The remaining greater discrepancies showed to be clustered in defined areas like the Zagros chain and the reliefs along the Caspian coastline and the East borders.

## 1 Introduction

The Mohorovičić discontinuity interface, representing the boundary where there are significant variations in body wave velocities within the Earth, is effectively and extensively determined through the examination of seismic activity originating from distant sources deep within the Earth. Despite the dependability of seismic exploration procedure in providing Moho characteristics, it grapples with formidable constraints pertaining to spatial resolution.

**Fig. 1:**
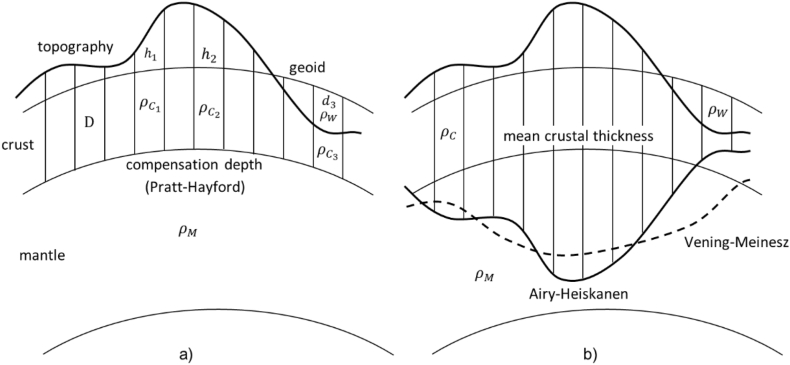
Sketch of models based on the concept of isostasy: the Pratt-Hayford model on the left side (figure a) and the Airy-Heiskanen and the Vening-Meinesz models on the right (figure b).

The presence of this boundary can be ascertained through the utilization of gravimetric-isostatic methodologies and gravity data in conjunction with the seismic techniques. It seems sense to assume that a reduction of topographic masses would lead to decorrelated gravity data when the crust is seen as a constant and uniform layer. Contrary to this assumption of decorrelated gravity observations due to a homogeneous crust, the pioneering measuring expedition of the meridian arc in Peru, conducted by Pierre Bouguer during the 18th century, uncovered that this was not the actual scenario [1]. During his measurements, Bouguer observed a notable discrepancy: the deflections of the vertical, as determined by his observations, were considerably smaller than what would be anticipated from the calculation of topographic attraction that are brought on by the Andes Mountains' presence. Later in 19th century George Everest also observed the same concept when surveying the Himalayan part of Northern India. Indeed, it was Bouguer's discoveries in Peru and Everest's views of the Himalayas that led scientists to realize that the effects of topographic masses needed to be somehow counterbalanced or compensated for inside the Earth's interior. The notion of isostasy was created and introduced as a result of this revelation. In line with this theory, an equilibrium can be reached at a particular amount of compensation. It resembles arriving at a place where everything calms down and achieves harmony [2] which

(1) $$\int_{D}^{h}\rho dr=cost$$

holds. It means that, there is a balance between the variations in density (ρ) within the Earth's interior, from the depth of compensation (D) to the topographic height (h), resulting in a consistent overall value. In the realm of geodesy, researchers have developed diverse isostatic models tailored to meet specific requirements. While these models may be simplified depictions of the intricate isostatic processes, they serve the valuable purpose of mitigating systematic effects within a remove-compute-restore (RCR) framework. By adopting this approach, geodetic measurements can be refined, ensuring a more accurate understanding of gravitational variations arising from surface features. In the following, the most prominent types of isostatic models, namely Pratt-Hayford, Airy-Heiskanen, and Vening Meinesz are described ([1,3], and [4]). The Airy-Heiskanen and Pratt-Hayford ideas, which assume that topographic masses only affect the vertical column underneath them, are restricted to local applications. Instead, being limited to simple localized impacts, surface loads are actually more likely to have a widespread impact. In this regard, the Airy-Heiskanen method has been modified by the Vening Meinesz model, which takes into consideration the elastic characteristics of the Earth's crust. According to this idea, the crust is elastic and is capable of bending as a result of topographic masses. As a result of the gravitational impacts of these masses, the Earth's surface is locally deformed or warped, as seen in Fig. 1b. With this adjustment, the Vening Meinesz model may now describe the gravity field and geodetic data more precisely, especially in areas with considerable topographic masses or geological features. The Vening Meinesz model provides a more thorough understanding of the intricate connections between gravity, topography, and the Earth's structure by taking into account the elastic behavior of the Earth's crust. A general formulation of this model can be found in Moritz's book [5]. The derivation of the bending curve of the crust is given for instance in Abd-Elmotaal's work [6]. For geodetic purposes, the Airy model is mostly considered as sufficient and therefore will also be used in this paper.

A constant compensation depth, designated as "D," is incorporated into the Pratt-Hayford gravity model's formulation. This compensatory depth is considered to have a set value that is commonly calculated from sea level on Earth and is roughly 100 km. The notion of this model is illustrated by Fig. 1a, which shows the compensation depth D as a calculational parameter. Above a certain depth, the masses are thought to be arranged in vertical columns. In order for each column to have the same mass, a density value is assigned to each one. An assumed homogeneous and consistent density distribution exists below this depth. For instance, in case of the absence of topography (h = 0), the according density value of this column is commonly introduced as $\rho_{0}$ = 2670 kg/m3, the standard crustal density. The general isostatic condition for a certain column $c_{i}$ with height $h_{i}$ above sea level is then given by

(2) $$\rho_{C_{i}}\left(D+h_{i}\right)=\rho_{0}D$$

while over ocean areas

(3) $$\rho_{C_{i}}\left(D-d_{i}\right)+\rho_{W}d_{i}=\rho_{0}D$$

where $d_{i}$ and $\rho_{W}$ = 1027 kg/m3 stands for the depth of the ocean of the corresponding column and the standard density of sea water respectively.

According to Airy-Heiskanen, the isostatic idea adopts a distinct viewpoint to comprehend the behavior of the Earth's crust and its connection to the subsurface mantle. The variation in density between the topographic load and the denser underlying material plays a crucial role in the isostatic equilibrium. The denser layer with a density of $\rho_{M}$ = 3270 kg/m^3^ beneath exerts an upward buoyancy force on the topographic load with a density of $\rho_{C}$ = 2670 kg/m^3^, counteracting the downward gravitational force caused by its mass. This balance of forces helps maintain isostatic equilibrium, influencing the thickness and distribution of the Earth's crust. The model is envisioned as resembling an iceberg, with the crustal material constituting the topography floating over the mantle, in order to attain isostatic stability. As a result, the crustal material sinks deeper into the mantle as the height of the topography rises. Fig. 1b depicts this behavior. Thus, isostatic equilibrium between penetration depth $t_{i}$ with respect to an assumed mean crustal thickness D and the topographic height $h_{i}$ for a certain column $C_{i}$ over land is found by

(4) $$\Delta \rho t_{i}=\rho_{C}h_{i}$$

with the density contrast between mantle and crust $\Delta \rho =\rho_{M}-\rho_{C}$. For ocean areas the relation is given by

(5) $$\Delta \rho t_{i}=\left(\rho_{C}-\rho_{W}\right)d_{i}$$

where again $d_{i}$ is the depth of the ocean and $\rho_{W}$ = 1027 kg/m^3^ represents the sea water standard density.

These conceptual frameworks are put out in the context of isostasy to explain the consistent disparities seen in geodetic measurements when compared to the forecasts based on the straightforward presumption that the Earth's core is equally stratified. An important innovation is presented, namely in the Airy-Heiskanen model (and also in the Vening Meinesz idea), incorporating the notion of a changing surface boundary separating the denser mantle from the less dense crust. Surprisingly, the Mohorovii discontinuity, or the Moho, can be understood as this border. This conceptual foundation serves as the foundation for the study's development of the Least Square Collocation (LSC) method, which has the potential to shed light on how to identify the elusive Moho discontinuity using superior GOCE (Gravity field and steady-state Ocean Circulation Explorer) gradient data. By utilizing the power of LSC, the inquiry aims to decipher the subtle linkages affecting the lithosphere and mantle interactions between gravity field changes, isostatic equilibrium, and the illusive Moho interface.

In the domains of geophysics and physical geodesy, the process of inferring subsurface density patterns from gravity data constitutes a typical ill-posed inverse problem [3]. The identification of precise density structures is difficult since such poorly stated issues lack distinctive answers. However, under certain proper assumptions, gravity data is often used in both forward and inverse geophysical modeling, despite this inherent difficulty. In forward modeling methodologies, the process begins with assuming internal density distributions and layers in an a-priori model. From this initial model, the resulting gravity effect is calculated. The calculated gravity values are then compared to the actual observed data. Subsequently, the density model is iteratively adjusted or changed in a step-by-step manner until the synthetic gravity values obtained from the model closely match the measured gravity values from the observations [7]. This iterative process allows geophysicists to refine the initial density model systematically until it converges to a solution that best fits the observed gravity data. By adjusting the density distribution in the subsurface, the model seeks to find the most plausible representation of the internal density structures that give rise to the observed gravity anomalies. Direct inversion techniques are used in the second class of methodologies for analyzing gravimetric data. This technique's foundation is a two-layer model with a constant density contrast between the two layers. Furthermore, it is assumed that the mean depth of the border surface between these two layers is known. It may be demonstrated that the inverse gravimetric problem has a unique solution under these particular presumptions ([[8], [9], [10]]).

Moritz introduced the Vening Meinesz isostatic concept [5], which enabled the derivation of Moho depths from isostatic gravity anomaly data in a global spherical approximation. Subsequently, Sjöberg [11] made further improvements by employing integral equations to address the inverse problem and infer subsurface density variations, leading to more accurate determinations of Moho depths. In Bagherbandi's study in 2011 [12], the application of the method was examined for estimating Moho depths using gravity gradient data similar to those provided by the GOCE mission. The research focused on regional investigations in Scandinavia and Iran, where the approach was tested and refined to infer the Moho depths accurately in these specific geographical areas. Using GOCE data with the preliminary seismic model CRUST2.0 [13,14] suggested an alternate technique for calculating the global Moho depth. This method required doing the inversion using spectral Wiener methods. In order to get more precise estimations of the Moho depth on a worldwide scale, the researchers combined the gravity data from the GOCE mission with the seismic model CRUST2.0. In the study done by Ref. [15], the approach used by Ref. [14] was later used in a regional setting. This regional application allows for a more concentrated research of particular locations, offering insightful knowledge regarding the tectonics and crustal structure of those places. Recently [16] proposed an application of the study done by Ref. [17] of a closed loop simulation of a collocation approach in planar approximation. In this paper a similar methodology has been investigated for the Moho estimate in a regional area based on GOCE data and seismic depths information.

### 1.1 The proposed methodology

In this paper, a method for using GOCE Satellite Gravity Gradiometer (SGG) data in the spatial domain is devised. The Least Squares Collocation (LSC) approach is the one used for this purpose. Based on observable data points and their spatial correlations, the LSC method is a mathematical approach for estimating unknown values. In the study, the GOCE SGG data are processed using the LSC technique, enabling the estimation of numerous geophysical characteristics and properties connected to the Earth's gravity field. This novel idea, which is comparable to the Airy-Heiskanen method, is based on the simplified two-layer isostatic model, like many of the research previously described. The idea of hydrostatic equilibrium is used over continental regions in this model. The topographic load of a mass column with height $h_{i}$ and depth $t_{i}$ are related by the formula $\Delta \rho t_{i}=\rho_{C}h_{i}$, which was previously used to explain this idea. The depth $t_{i}$ is a representation of the amount by which the topographical load is causing the Earth's crust to sink into the mantle material.

The inversion approach implicitly depends on two crucial hypotheses to guarantee a unique and trustworthy result. First, we assume that the densities of the crustal material ($\rho_{C}$) above and the mantle material ($\rho_{M}$) under the Moho surface are constant throughout the process. This implies that the density contrast, or difference in density between mantle and crustal materials ($\Delta \rho =\rho_{M}-\rho_{C}$), also stays unchanged. In other words, we assume these layers as homogeneous and ignore any density differences along them. The second and crucial step is that we measure the depths ($t_{i}$) in respect to a known average crustal thickness (D). We may regard the depths as deviations or variations from the standard value because of this presumption, which we established before performing the inversion.

**Fig. 2:**
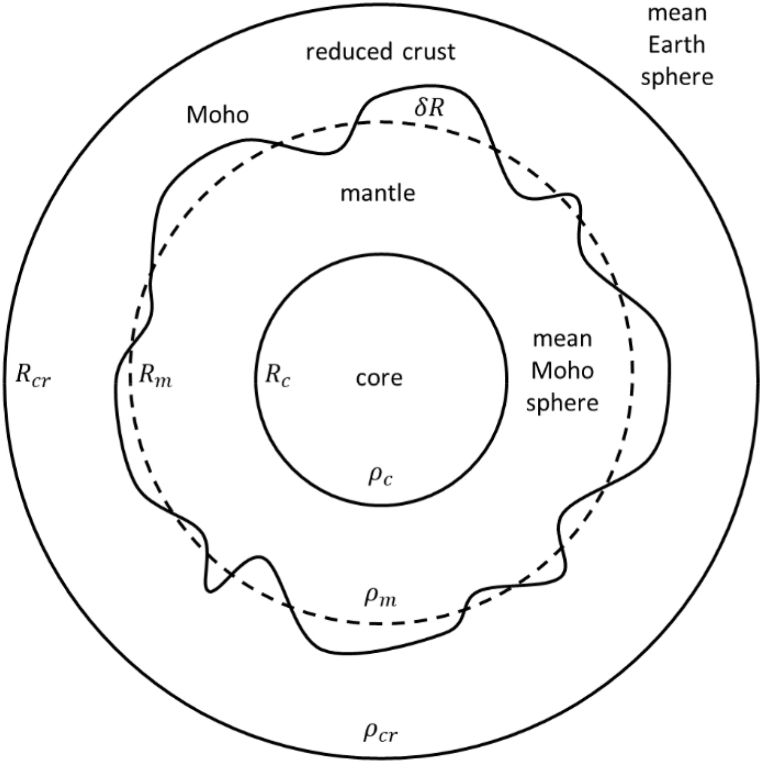
Sketch of the simplified two-layer isostatic model of the Airy-Heiskanen type. $R_{c}$ , $R_{m}$ and $R_{cr}$ are the radius of the spherical shells representing core, mantle and crust with constant densities of $\rho_{c}$, $\rho_{m}$ and $\rho_{cr}$, respectively.

The isostatic notion also exposes another important realization. With the exception of lateral fluctuations brought on by the Moho border, we assume that the density distribution in the interior of the Earth is constant. According to this argument, any differences in the topographic masses and the resulting isostatic adjustment are the only causes of changes in the external gravity field. Mountains and deep basins are examples of mass differences that cause changes in the isostatic equilibrium inside the Earth's crust, which eventually affects the externally observable gravity field. We may also take into account the underlying atmospheric masses because the GOCE observations take place in outer space at a height of around 250 km, but this contribution would be comparatively insignificant. The residual gravity field that results from removing the gravitational effects of topographic and atmospheric masses from the gravity data should solely represent the remaining isostatic compensation effect, providing information about the Moho fluctuations [18]. In fact, a simplified Earth model can be the one based on three shells representing core, mantle and crust (Fig. 2).

The potential of this body outside the mean Earth sphere is

(6) $$T\left(P\right)=G\left[\frac{4}{3}\pi \frac{R_{c}^{3}\rho_{c}}{r}+\frac{4}{3}\pi \frac{{(R}_{m}^{3}-R_{c}^{3}{)\rho }_{m}}{r}+\frac{4}{3}\pi \frac{{(R}_{cr}^{3}-R_{m}^{3}{)\rho }_{cr}}{r}+\left(\rho_{m}-\rho_{cr}\right)\int_{S_{m}}\int_{R_{m}}^{R_{m}+\delta R}\frac{1}{l}dv\right]$$

with $R_{c}$ = core radius, $R_{m}$ = Moho mean radius*,* $R_{cr}$ = mean Earth radius*,* G = gravitational constant. So, by reducing in the data (that are given on and outside $R_{cr}$) the topographic effect, compensating for density anomalies in the crust and evaluating T(P) on the mean Earth sphere so that ${r=R}_{cr}$, or on any other concentric spherical surface having radius greater than $R_{cr}$, one has

(7) $$T\left(P\right)=const.+G\left(\rho_{m}-\rho_{cr}\right)\int_{S_{m}}\int_{R_{m}}^{R_{m}+\delta R}\frac{1}{l}dv=const.+G\Delta \rho \int_{S_{m}}\int_{R_{m}}^{R_{m}+\delta R}\frac{1}{l}dv=const.+\delta T$$

in the assumption that signals coming from deeper mass anomalies can be considered as constant at least local level, that is in the investigation presented in this paper. Then, subtracting the *const*. from the data, one is left with the last term only. Using a Helmert condensation approach, the δT term can be represented as the potential of a single layer on the mean Moho sphere $S_{m}$ of radius $R_{m}$.

(8) $$\delta T=G\int_{S_{m}}\frac{\rho_{S}}{l}ds$$

The relationship giving the surface density *ρ*_*S*_ can be obtained by assuming mass conservation between the Moho column of height δR and the mass in the condensation layer over the same elementary area element [19]

(9) $$\Delta \rho d\sigma \int_{R_{m}}^{R_{m}+\delta R}r^{2}dr=\rho_{S}R_{m}^{2}d\sigma$$

where dσ is the infinitesimal surface element of the unit sphere. By exploiting (9) we then find

(10) $$\rho_{S}=\Delta \rho \frac{{\left(R_{m}+\delta R\right)}^{3}-R_{m}^{3}}{3R_{m}^{2}}$$

Then, by following the approach described in Ref. [2], one can derive the following equation

(11) $$\delta T\left(P_{m}\right)=\frac{{2R}_{m}}{3}[2\pi G\rho_{S}\left(P_{m}\right)-\delta \Delta g\left(P_{m}\right)]$$

giving the “anomalous potential” $\delta T\left(P_{m}\right)$ as a function of the surface density $\rho_{S}\left(P_{m}\right)$ and of the “gravity anomaly” $\delta \Delta g\left(P_{m}\right)$ related to $\delta T\left(P_{m}\right)$. Inserting the equation

(12) $$\delta \Delta g=-\frac{\partial \delta T}{\partial r}-2\frac{\delta T}{r}$$

which formally links $\delta \Delta g\left(P_{m}\right)$ to $\delta T\left(P_{m}\right)$, one can obtain

(13) $$\delta T\left(P_{m}\right)=\frac{{2R}_{m}}{3}[2\pi G\rho_{S}\left(P_{m}\right)+{\frac{\partial \delta T}{\partial r}|}_{r=R_{m}}+2\frac{\delta T}{R_{m}}\left(P_{m}\right)]$$

and finally

(14) $$\rho_{S}\left(P_{m}\right)=-\frac{1}{4\pi G}\left[{2\frac{\partial \delta T}{\partial r}|}_{r=R_{m}}+\frac{\delta T}{R_{m}}\left(P_{m}\right)\right]$$

This is the linear operator linking the surface layer density $\rho_{S}$ to the potential δT(P).

The collocation approach for the estimate of δR.

Following [20], we assume that the covariance function of the potential δT(P) has the form

(15) $$C_{\delta T\delta T}\left(r_{P},r_{Q},\psi_{PQ}\right)=\sum_{n=2}^{+\infty }{\left(\frac{R_{B}^{2}}{r_{P}r_{Q}}\right)}^{n+1}k_{n}P_{n}\left(\cos\;\psi_{PQ}\right)$$

Assuming further that we have observations of ${\delta T}_{rr}=\frac{\partial^{2}\delta T}{\partial r^{2}}$ and considering equation (14), by covariance propagation law [21] we have

(16) $$C_{\rho_{s}\delta T_{rr}}\left(P,Q\right)=-\frac{1}{4\pi G}\left(2\frac{\partial }{\partial r_{P}}+\frac{1}{r_{P}}\right)\left(\frac{\partial^{2}C_{\delta T\delta T}\left(P,Q\right)}{\partial r_{Q}^{2}}\right)$$

Furthermore

(17) $$C_{{\delta T}_{rr}\left(P\right){\delta T}_{rr}\left(Q\right)}=\left(\frac{\partial^{2}}{\partial r_{P}^{2}}\frac{\partial^{2}C_{\delta T\delta T}\left(P,Q\right)}{\partial r_{Q}^{2}}\right)$$

In explicit form, by applying (16) and (17) to (15), we get

(18) $$C_{\rho_{s}\delta T_{rr}}\left(P,Q\right)=\frac{1}{4\pi G}\sum_{n=2}^{+\infty }{\left(\frac{R_{B}^{2}}{r_{P}r_{Q}}\right)}^{n+1}\frac{\left(2n+1\right)\left(n+1\right)\left(n+2\right)}{r_{P}r_{Q}^{2}}k_{n}P_{n}\left({\cos\;\psi }_{PQ}\right)$$

(19) $$C_{{\delta T}_{rr}{\delta T}_{rr}}\left(P,Q\right)=\sum_{n=2}^{+\infty }{\left(\frac{R_{B}^{2}}{r_{P}r_{Q}}\right)}^{n+1}\frac{{\left(n+1\right)}^{2}{\left(n+2\right)}^{2}}{r_{P}^{2}r_{Q}^{2}}{k_{n}P}_{n}\left(\cos\;\psi_{PQ}\right)$$

According to the Wiener-Kolmogorov theory the collocation estimator of $\rho_{s}$ is

(20) $$\rho_{s}=C_{\rho_{s}\delta T_{rr}}{\left(C_{\delta T_{rr}\delta T_{rr}}+C_{nn}\right)}^{-1}{\delta T}_{rr}$$

Then, by using (10) we obtain

(21) $$R_{m}+\delta R=\sqrt[{3}]{\frac{3\rho_{S}R_{m}^{2}+\Delta \rho R_{m}^{3}}{\Delta \rho }}$$

or, disregarding terms of the order of $\frac{\delta R^{2}}{R_{m}}$ in (10)

(22) $$\delta R=\frac{\rho_{S}}{\Delta \rho }$$

For a given density contrast Δρ and a given mean Moho radius $R_{m}$, equation (21) or (22) are the equations solving the Moho estimate problem for the devised model.

### 1.2 The case study: the Iranian plateau area

**Fig. 3:**
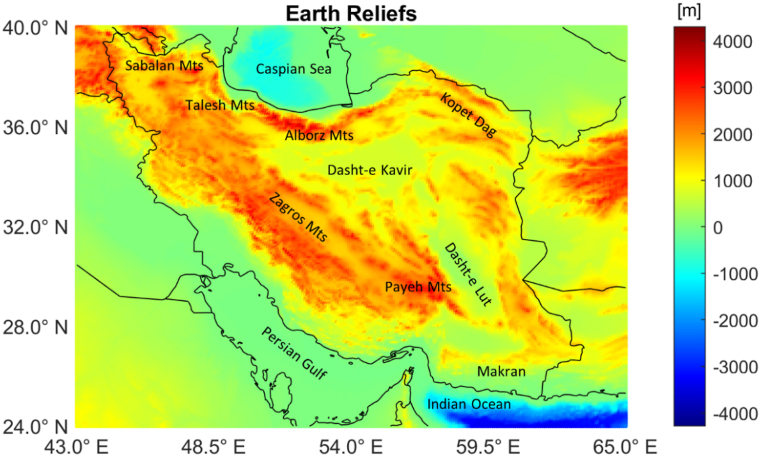
The Iranian Plateau and its main reliefs.

The Iranian plateau, detailed in Fig. 3, has been selected as test case for the proposed approach.

The existence of subduction (Makran in the southeast) and collision zones (Zagros orogeny in the southwest), volcanic areas (Damavand in the north, Sabalan in the northwest, the active volcano Taftan in the southeast and Bazman in southwest of Taftan Peak), deserts (Dasht-e Kavir and Dasht-e Lut in the central area), and seas (Caspian to the North and both the Persian Gulf and the Indian Ocean to the South), make the Persian/Iranian plateau a unique place as a natural laboratory with various tectonic settings. This tableland, which is an active tectonic regime of the Alpine–Himalayas sandwiched between the Arabian and the Eurasian Plates, was created as a result of the Tethys Ocean closing and the collision of the Arabian and Eurasian plates during the Cenozoic age, which led to the active and recent tectonic structures indicated above. Due to the variety and complexity of crustal deformations, the actual formation of the Iranian Plateau's lithosphere structure, including Moho, with different modes of tearing and shearing, is currently being debated.

Among the various studies and research regarding the Moho depth over the Iranian plateau over the past twenty years, three main branches can be identified, i.e., seismic methods, approaches based on the gravity-isostasy concept, studies integrating seismic/gravity information. In general, there is agreement among these studies about the places where the crust is thicker or thinner. However, some discrepancies (sometimes of the order of tenth of km) in some areas are unavoidable because of using different data, methods, hypotheses and assumptions, proving how much the Iranian Plateau is a challenging test case.

A study of [22] based on Receiver function (RF) technique estimated crustal thicknesses of 50 km for KAR, 47 km for ZOW, and 44 km for HAM stations in the Kopeh-Dagh region in the Northeast Iran [23]. estimated the thickness of the Lithosphere–Asthenosphere Boundary (LAB) beneath the Central Alborz region by P- and S-receiver function methods while [24] investigated the lithospheric structure beneath Northwest Iran by implementing the Zhu and Kanamori's approach [25]. [26] is another study that did the same but for whole areas of the Iranian tectonic block. The maximum Moho depth was estimated along the Zagros Mountains belt beneath the Sanandaj-Sirjan Zone (SSZ); however, the thinnest crust of about 33 km is estimated beneath the Makran subduction zone. Also [27] estimated the Variation of the Moho depth and Vp/Vs ratio beneath Central Iran, Eastern Iran, and Makran regions using receiver functions analysis. They estimated the maximum depth of 64 km for the NGRK station in the Zagros belt and a minimum of 30 km for the TNSJ station south of Dasht-e Kavir [28]. analyzed the velocity structure of the central Alborz Mountains in the north of Iran and estimated the minimum and maximum of 46 km and 58 km for Moho within the study area and concluded there is a moderate root but insufficient to compensate the height of the mountain. Moreover [29], used simultaneous inversion of data from RFs and fundamental mode Rayleigh wave group velocity to obtain high-resolution structures of the lithosphere system beneath a seismic profile in Iran. Other studies in this notation are research done by Ref. [30] in Alborz Mountains and [31] in the Makran subduction zone.

Mousavi and Fullea [32] investigated the 3-D crustal structure of the Iranian plateau by integrated geophysical–petrological modeling combining elevation, gravity, and gravity gradient fields, seismic and petrological data. They estimated the maximum Moho depth equal to 65 km beneath the Zagros Mountain, while the thinnest crust was reported for the Oman sea and the central part of Iran [33]. presented two Moho models using terrestrial and Earth Geopotential Model (EGM) for Iran with the Root Mean Square (RMS) of 2.7 km. They compared their models with the CRUST2.0 global Moho model, and RMS was in the order of 4.15 and 3.45 km for terrestrial and EGM data, respectively [16]. investigated three gravimetric methods, namely, collocation, Jeffrey, and Sjöberg, using the GOCO03S model to construct Bouguer gravity anomaly reduced by topography/bathymetry, sediment, and consolidated crust effects. They compared the result with point-wise seismic studies and estimated a standard deviation of around 6 km. Overall, they concluded that their result was in an acceptable range with respect to the seismic data except for Makran, Persian Gulf, and Caspian Sea areas which can be addressed by the poor quality of CRUST1.0 data in these areas [34]. implemented the collocation approach to invert the GOCE gradient data for the estimation of Iranian Moho in the frequency domain, which overcame one of the biggest weak points of LSC, which is computationally expensive because of its covariance matrixes. They compared the achieved map of Moho with active faults in the study area and asserted that Moho features could be directly associated with geophysical and tectonic blocks.

In the context of the aforementioned studies, the proposed approach for Moho reconstruction in the Iranian plateau area has been applied and compared.

### 1.3 Gravity data reduction

As observed data, radial second derivatives of the disturbing potential $T_{rr}$ were computed on a regular spherical grid in the area spanning 20.5°–44.5° N in latitude and 39.0°–69.0° E in longitude with a grid spacing of 0.15°. In particular, GOCO06s [35] model was considered as reference model for data synthesis at full resolution up to degree 300 but neglecting the longer wavelengths. In fact, as stated in Ref. [11] the long-wavelength contribution to the gravity field is mainly due to deep mantle density variations and this implies that the long wavelengths of gravity (approximately up to degree and order 10), would lead to systematic errors of the computed Moho topography. For this reason the first 10° were excluded from the data synthesis. The radius of the observation grid was set to 6591 km that is compatible with the original satellite mission altitude of approximately 220 km in terms of WGS84 ellipsoidal height.

According to the followed approach based on the concentric spherical shells at constant density approximating the whole Earth mass, observation data were reduced for topographic and sediments masses. In this regard, the latest GEBCO_2021 Grid was considered as a reference model for surface elevations and bathymetry and the CRUST1.0 [36] model for sediment thickness. Due to computational constraints, for the scope of both neglecting far field effects and to compute mass correction in spherical approximation so to be consistent with the observation data, these topography, bathymetry and sediments effects were recomputed on a global common grid of 5’ and analyzed in terms of spherical harmonics coefficients up to d/o 720.

**Fig. 4:**
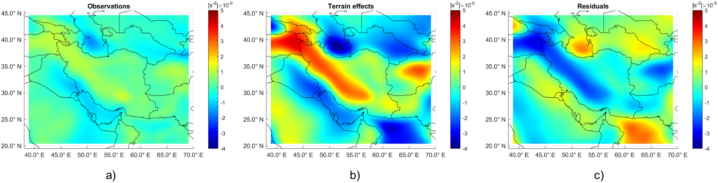
Plots of the observation data (figure a), the combined terrain effects of topography, bathymetry and sediments (figure b) and residual data (figure c). All plots are expressed in terms of $T_{rr}$ and $\frac{1}{s^{2}}$ units.

Topographic contribution was then computed on the basis of such coefficients, synthetized for d/o in the range 10–300 for consistency with the observation data and at the same location. Data reduction was then applied by properly correcting observation data of the contributes in terms of $T_{rr}$ of crust, sea and sediments with densities of 2670, 1030 and 2400 kg/m^3^, respectively. Fig. 4 shows the observed data (a), the terrain correction (b) and the subsequent residual data (c).

From the obtained residuals, it can be noted the great variability of the gravity signal on the study area and the clear signature of local features across the plateau and the southern Caspian Sea.

### 1.4 The Moho collocation estimate

The empirical covariance of the gridded residuals was computed up to a spherical distance of 12° with a sampling of 0.75°. The covariance model fitting was then performed on the basis of such values by properly tuning the parameters of the well-known Tscherning-Rapp models [37] propagated to the observed functional of the anomalous potential, i.e., $T_{rr}$. Tests have been carried out to verify which Tscherning-Rapp model was more suitable for covariance fitting. The best fit covariance model function that was selected is

(23) $$C_{{\delta T}_{rr}\left(P\right)\delta T_{rr}\left(Q\right)}=\sum_{n=10}^{+300}{\left(\frac{R_{B}^{2}}{r_{P}r_{Q}}\right)}^{n+1}\frac{{\left(n+1\right)}^{2}{\left(n+2\right)}^{2}}{r_{P}^{2}r_{Q}^{2}}\frac{A}{\left(n-1\right)\left(n-2\right)\left(n+B\right)}P_{n}\left(\cos\;\psi_{PQ}\right)$$

**Fig. 5:**
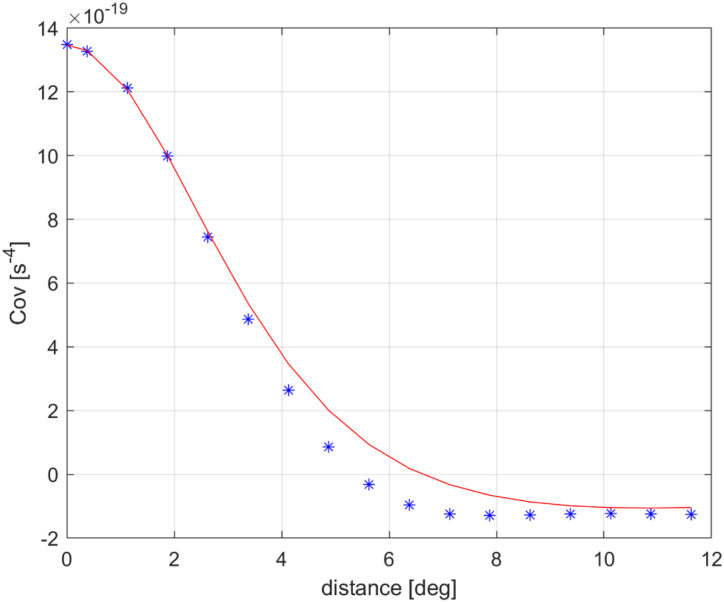
Empirical covariance and covariance model fitting.

The Bjerhammar sphere radius $R_{B}$ was set to 100 km below the mean Earth radius which was assumed to be 6378 km. Fig. 5 shows the covariance model fitting obtained by setting the $\hat{A}$ parameter equal to 68x10^5^. and $\hat{B}$ to 24. As it can be noted, the agreement is good in the range 0°–4° where empirical values are expected to be more reliable.

The auto-covariance function of the $\delta T_{rr}$ values was then defined as

$$C_{{\delta T}_{rr}\left(P\right)\delta T_{rr}\left(Q\right)}=\sum_{n=10}^{+300}{\left(\frac{R_{B}^{2}}{{\bar{R}}_{obs}^{2}}\right)}^{n+1}\frac{{\left(n+1\right)}^{2}{\left(n+2\right)}^{2}}{{\bar{R}}_{obs}^{4}}\frac{\hat{A}}{\left(n-1\right)\left(n-2\right)\left(n+\hat{B}\right)}P_{n}\left({\cos\;\psi }_{PQ}\right)$$

with ${\bar{R}}_{obs}$ = 6591 km. The model covariance function indicated an almost negligible observation noise, of the order of about 1 % of the power of the modelled signal and used for the determination of $C_{nn}$. According to (18), the cross-covariance model of $C_{\rho_{s}\delta T_{rr}}$ was set as

$$C_{\rho_{s}\left(P\right)\delta T_{rr}\left(Q\right)}=\frac{1}{4\pi G}\sum_{n=10}^{+300}{\left(\frac{R_{B}^{2}}{R_{m}{\bar{R}}_{obs}}\right)}^{n+1}\frac{\left(2n+1\right)\left(n+1\right)\left(n+2\right)}{R_{m}^{2}{\bar{R}}_{obs}^{2}}\frac{\hat{A}}{\left(n-1\right)\left(n-2\right)\left(n+\hat{B}\right)}P_{n}\left({\cos\;\psi }_{PQ}\right)$$

Prediction points were located on a regular spherical grid in the area 25.5°–39.5° N and 44.0°–64.0° E, with a grid spacing of 0.1, lying on the surface of the sphere of radius $R_{m}$ (see equation (6)). The value of $R_{m}$ was set to 6330 km that corresponds to 48 km below the mean Earth radius. This value was selected as first guess of the mean Moho depth over the study area based on what can be found in literature. In particular, it was decided to use as reference the mean Moho depth reported by the Crust1 model in the area.

**Fig. 6:**
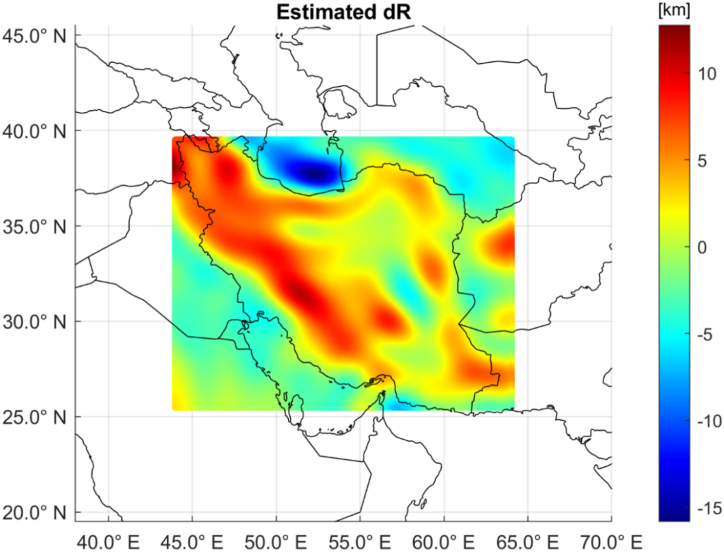
Plots of the estimated Moho depth variation. Positive numbers reflect sinking crust and Moho deepening.

By means of the collocation formula, $\rho_{s}$ was then computed by equation (20). Finally, the Moho depth variation δR was obtained by equation (21), assuming that the density contrast between mantle and crust $\Delta \rho =\rho_{M}-\rho_{C}$ is Δρ=600 kg/m^3^. In Fig. 6, the estimated δR values are plotted in the area under investigation.

The estimated Moho depth variation ranges approximately between −15 km and +12 km with a clear maximum in the southern Caspian Sea and minima that are aligned with the main Iranian reliefs like the Alborz Mts at North, the Zagros Mts along the South-West borders chain, the Sistan and Baluchestan province in the South-East and the Kerman area in the Central Iran.

**Fig. 7:**
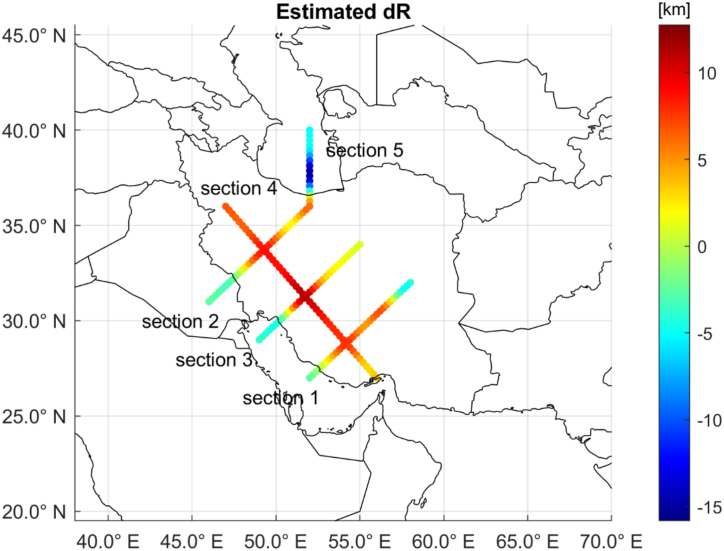
Plots of the estimated Moho depth variation along representative sections. Positive numbers reflect sinking crust and Moho deepening.

Such main features were better analyzed along representative sections, displayed in Fig. 7, and compared with similar computations based on the approach followed in Ref. [38].

**Fig. 8:**
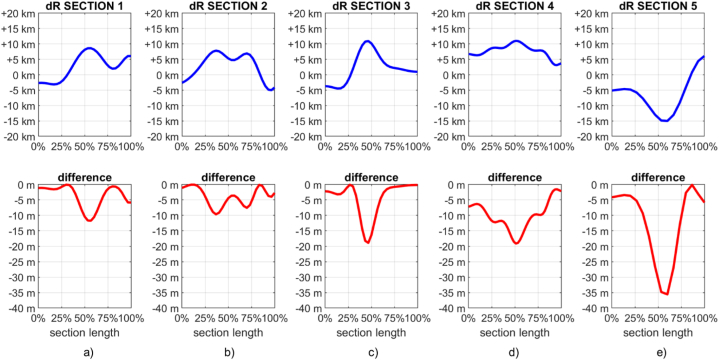
Estimated Moho depth variations along the analyzed profiles (top pictures) and differences between the estimates obtained with the current proposed approach and those obtained with the one described in Ref. [38] (bottom pictures). Note the different orders of magnitude and how no differences are obtained when $\hat{\delta R}$ = 0.

As it can be seen from the plots of Fig. 8 (a-e), the two different approaches (that were based on the same data and the same covariance parameters) lead to almost equivalent results with small differences that are four orders of magnitudes smaller than the target quantity which is of the order of tenths of km. However, it has to be remarked that no difference is obtained at $R_{m}$ (i.e., when $\hat{\delta R}$ = 0) and that the two approaches diverge as $\left|\hat{\delta R}\right|$ increases. Also, it can be seen that the current proposed approach leads to smoother results than those obtained with the method in Ref. [38].

### 1.5 Tuning the Moho gravity estimate with seismic information

**Fig. 9:**
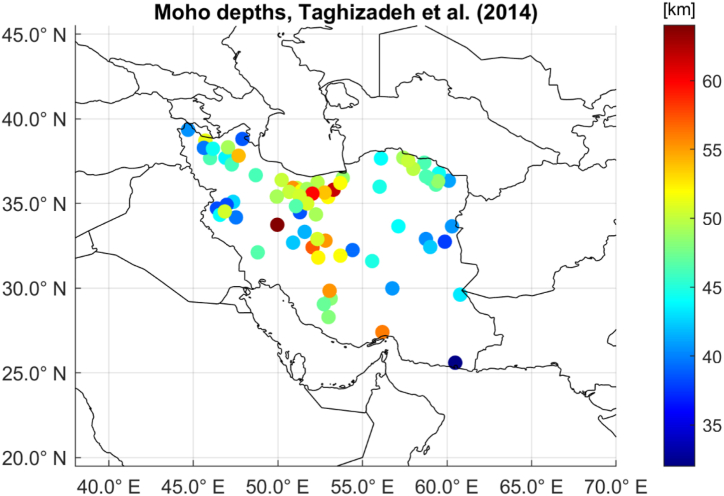
Plots of the Moho depths based on seismic information as reported in Table 1 of [26] and taken as benchmark values.

As previously described, the computed Moho depth variations are based on some a priori assumptions, particularly the reference Moho radius $R_{m}$ and the density contrast Δρ. Based on what has been found in literature, such assumptions can be revised and refined, according to seismic studies. In this sense, the previously presented extensive literature review was taken as reference and integrated in the estimation procedure for guiding the estimation procedure. In fact, based on this literature review, Moho depths reported in the work of [26] were used as benchmark estimates for determining suitable average Moho depth average density contrast in the investigated area. This work was selected because it is based on a significant number of seismic stations, well distributed on the whole area. Moreover, estimated Moho depths are based on three different methods and comparisons among them are discussed. Fig. 9 shows the considered seismic stations and the estimated Moho depths.

These benchmark values were obtained by averaging three Moho depths for each station, computed with different techniques. With respect to the original dataset, three stations were not considered (ASAO, RMKL and AHRM) being their Moho depths determined with two available out of three techniques only. Moho depths were reported at station position as latitude, longitude and altitude without details on the adopted reference systems: in the computations they were assumed as WGS84 latitude and longitude. Regarding the altitude, the reported heights were assumed to be orthometric heights, converted to ellipsoidal heights by means of EGM2008 geoid values. Furthermore, SHI station reported an anomalous altitude (almost 16 km, clearly a typo error) it was then rejected from the benchmark dataset that was finally composed of 73 stations.

**Fig. 10:**
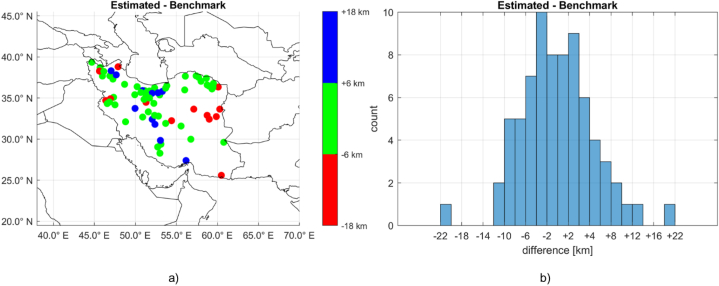
Differences between the refined solution obtained with the proposed approach based on gravity-only data and the seismic-based benchmark values in terms of spatial distribution (figure a) and histogram of the values (figure b).

In order to properly compare the seismic derived Moho depths with the proposed approach in terms of $R_{m}+\delta R_{m}$, the seismic depths were assumed to be referred to the station ellipsoidal heights. Then, the seismic $R_{moho}$ have been obtained as the ellipsoidal heights of the stations minus the reported depths. Finally, they were converted into spherical coordinates to be coherent with the gravimetric estimated Moho depths. In order to find the $R_{m}$ value coherent with the sparse seismic $R_{moho}$, we applied the devised procedure varying the assumed mean Moho depth for a fixed Δρ value. This was done with an empirical procedure that still assumed Δρ equal to a reasonable value of 600 kg/m^3^ and iterated the estimation procedure with different values of $R_{m}$ ranging from 65 km to 40 km with steps of 1 km below the mean Earth radius of 6378 km. With such procedure, it was found the best fit with $R_{m}$ = 6321 km. The statistics of the residuals with respect to the benchmark $R_{moho}$ values were: μ = 0.299 km, σ = 6.848 km, min = −22.039 and max = +21.113 km. As second step, a further refinement to the set of ${\hat{R}}_{moho}$ values was done by fixing the value of $R_{m}$ to 6321 km and iterating on the assumed Δρ in the range 400 and 800 kg/m^3^ with steps of 0.5 kg/m^3^. The best results were obtained with Δρ equal to 650 kg/m^3^ and the statistics of the residuals on the benchmark $R_{moho}$ values were: μ = 0.079 km, σ = 6.749 km, min = −21.957 and max = +20.367 km. Fig. 10 shows the spatial distribution of the obtained classified residuals (a) and their distribution histogram (b).

As it can be noted, apart two stations (RAZ and CHBR), the great majority of the estimated residuals (66 %) fall in the range ±6 km that is comparable to the expected 3σ range of the benchmark values (having, from literature, accuracies of about 2 km). With respect to this 3σ range, 16 % of them resulted underestimated (yellow points in the figure) and clustered in the Alborz region and sparse along the Zagros chain while the remaining 18 % resulted overestimated (dark blue points in the figure), with a clear cluster in the reliefs of the Iran/Afghanistan border and other sparse points.

## 2 Summary and conclusions

This paper dealt with the estimate of the depth of the Mohorovicic discontinuity at regional scale. A simplified two-layer isostatic model of the Airy-Heiskanen type was considered and Earth was assumed to be composed by concentric spherical shells with homogeneous density distribution. Thus, the external gravity is considered as varying due to the changing topographic masses and the corresponding isostatic compensation. Least Squares Collocation approach was applied to retrieve the depth variation of the crust-mantle interface with respect to a reference spherical surface replicating the mean Moho depth at $R_{m}$. As observed data, GOCE model radial second derivatives of the disturbing potential $T_{rr}$ were considered at satellite height (about 220 km) and related to surface density $\rho_{S}$ on $R_{m}$ so to be able to give an estimate of δR given the density contrast Δρ between crust and mantle. The proposed approach was tested in the Iran area, being the Iranian Plateau a challenging test case due to the variety and complexity of crustal deformations and lithosphere structure.

First, the proposed approach was compared to a similar approach presented in a previous work. The obtained solutions were compared along six sections of the Zagros chain and in the Caspian Sea. They showed almost perfect consistency, even though the presented approach leads to slightly smoother results than the one applied in Ref. [38]. Furthermore, a refined solution was also proposed based on seismic estimates used as benchmarks. The seismic Moho values listed in Ref. [26] were selected for a numeric test. Given the values on 73 seismic stations, refinements on $R_{m}$ and Δρ were performed and improved statistics were obtained. The final standard deviation of the residuals between the gravity estimates of the Moho depths and the seismic derived values was found to be of about 3 km.

## CRediT authorship contribution statement

**Hadi Heydarizadeh Shali:** Writing – review & editing, Writing – original draft, Project administration, Investigation. **Carlo Iapige De Gaetani:** Visualization, Validation, Supervision, Software, Formal analysis. **Riccardo Barzaghi:** Supervision, Project administration, Methodology, Investigation, Formal analysis, Conceptualization. **Sabah Ramouz:** Writing – review & editing, Supervision, Investigation, Conceptualization. **Abdolreza Safari:** Validation, Supervision, Resources, Conceptualization. **Barbara Betti:** Validation, Investigation, Formal analysis. **Zohreh Abbasi Hafshejani:** Writing – review & editing, Writing – original draft, Visualization.

## Declaration of competing interest

The authors declare that they have no known competing financial interests or personal relationships that could have appeared to influence the work reported in this paper.
